# Acoustic radiation force impulse elastography, FibroScan^®^, Forns’ index and their combination in the assessment of liver fibrosis in patients with chronic hepatitis B, and the impact of inflammatory activity and steatosis on these diagnostic methods

**DOI:** 10.3892/mmr.2015.3299

**Published:** 2015-02-04

**Authors:** DAO-RAN DONG, MEI-NA HAO, CHENG LI, ZE PENG, XIA LIU, GUI-PING WANG, AN-LIN MA

**Affiliations:** 1 Department of Infectious Disease, China-Japan Friendship Hospital, Beijing 100029, P.R. China; 2 Department of Ultrasound, China-Japan Friendship Hospital, Beijing 100029, P.R. China; 3 Department of Ultrasound, Beijing University First Hospital, Beijing 100034, P.R. China; 4 Department of Pathology, China-Japan Friendship Hospital, Beijing 100029, P.R. China

**Keywords:** acoustic radiation force impulse, transient elastography, liver fibrosis, inflammatory activity, steatosis

## Abstract

The aim of the present study was to investigate the combination of certain serological markers (Forns’ index; FI), FibroScan^®^ and acoustic radiation force impulse elastography (ARFI) in the assessment of liver fibrosis in patients with hepatitis B, and to explore the impact of inflammatory activity and steatosis on the accuracy of these diagnostic methods. Eighty-one patients who had been diagnosed with hepatitis B were recruited and the stage of fibrosis was determined by biopsy. The diagnostic accuracy of FI, FibroScan and ARFI, as well as that of the combination of these methods, was evaluated based on the conformity of the results from these tests with those of biopsies. The effect of concomitant inflammation on diagnostic accuracy was also investigated by dividing the patients into two groups based on the grade of inflammation (G<2 and G≥2). The overall univariate correlation between steatosis and the diagnostic value of the three methods was also evaluated. There was a significant association between the stage of fibrosis and the results obtained using ARFI and FibroScan (Kruskal-Wallis; P<0.001 for all patients), and FI (t-test, P<0.001 for all patients). The combination of FI with ARFI/FibroScan increased the predictive accuracy with a fibrosis stage of S≥2 or cirrhosis. There was a significant correlation between the grade of inflammation and the results obtained using ARFI and FibroScan (Kruskal-Wallis, P<0.001 for all patients), and FI (t-test; P<0.001 for all patients). No significant correlation was detected between the measurements obtained using ARFI, FibroScan and FI, and steatosis (r=−0.100, P=0.407; r=0.170, P=0.163; and r=0.154, P=0.216, respectively). ARFI was shown to be as effective in the diagnosis of liver fibrosis as FibroScan or FI, and the combination of ARFI or FibroScan with FI may improve the accuracy of diagnosis. The presence of inflammatory activity, but not that of steatosis, may affect the diagnostic accuracy of these methods.

## Introduction

Hepatitis B is a global chronic disease caused by the hepatitis B virus (1). One consequence of infection with the hepatitis B virus is the development liver fibrosis, which can determine the prognosis as well as the therapy that is required (2). Fibrosis may progress to cirrhosis, which is an irreversible condition that may be viewed as the terminal stage of hepatitis. However, with the exception of a biopsy, there are currently no reliable indicators of the degree of liver fibrosis. Biopsies are invasive and there is a risk of serious complications (in up to 0.4% of cases). Furthermore, the results vary due to sampling errors, and intraobserver and interobserver variability (3). Therefore, the development of noninvasive examination methods is required, including real-time elastography, transient elastography (TE) and acoustic radiation force impulse elastography (ARFI) (4).

Liver stiffness measurement (LSM) using TE (FibroScan^®^) is accurate in identifying significant fibrosis, and in particular cirrhosis, in a number of liver diseases (5). This system is equipped with a probe consisting of an ultrasonic transducer mounted on the axis of a vibrator. A vibration of mild amplitude and low frequency is transmitted from the vibrator to the tissue by the transducer. This vibration induces an elastic shear wave which propagates through the tissue. At the same time, pulse-echo ultrasonic acquisitions are performed in order to follow the propagation of the shear wave and measure its velocity, which is directly associated with tissue stiffness (or elastic modulus). The harder the tissue, the faster the shear wave propagates. Recently, a study has shown that liver stiffness measurement using FibroScan allows the accurate prediction of hepatic fibrosis in patients with chronic hepatitis C virus infection (6). However, the cut-off values for different histological stages vary substantially between studies, patient groups and the aetiology of liver disease (5,6).

Recent studies have proposed that liver stiffness measurement may be conducted using ARFI elastography as a novel, reliable and accurate noninvasive approach to the evaluation of liver fibrosis (7). Studies have analyzed the performance of ARFI (8–13), although a number of these reports were heterogeneous, with small cohorts of patients and, in certain cases, without confirmation by liver biopsy. The combination of non-invasive tests for fibrosis may circumvent these limitations while improving diagnostic accuracy and resolving the discordances between tests (14). In this setting, Boursier and Cales (15) have proposed that a combination of liver stiffness evaluation (LSE) and blood tests for fibrosis may improve the diagnostic accuracy in patients with chronic hepatitis C. However, in a separate study, Castéra *et al* (16) reported that a combination of LSE and blood test did not improve the accuracy with which cirrhosis was diagnosed, although only a small number of blood tests was used, which were not contemporaneous. Whether the combination of ARFI and additional serological markers improves the diagnostic accuracy, thereby reducing the requirement for liver biopsy in patients with hepatitis B, has remained to be determined.

The Forns’ index (FI) is based on the platelet count, γ-glutamyl transpeptidase, age and cholesterol levels. The presence of significant fibrosis was predicted with a 96% negative predictive value (NPV) and 66% positive predictive value (PPV) using this method (17).

The aim of the present study was to assess the diagnostic accuracy of ARFI, FibroScan and FI, and to explore their combined effectiveness in evaluating liver fibrosis, with biopsy samples as the reference standard. In addition, the impact of inflammatory activity and steatosis on these diagnostic methods was investigated.

## Materials and methods

### Patients

**Figure 1:**
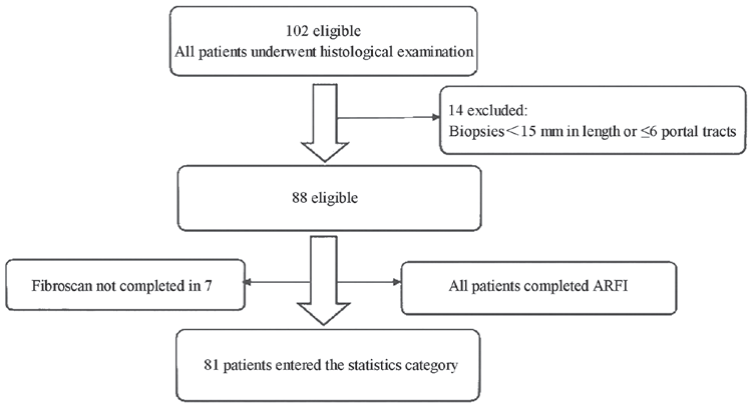
Flow diagram depicting patient assessments in the present study. ARFI, acoustic radiation force impulse elastography.

The present study comprised 81 consecutive patients with chronic hepatitis B (CHB), who had been admitted to the China-Japan Friendship Hospital (Beijing, China) from January 2011 to April 2013 (Fig. 1). The study was conducted in accordance with the declaration of Helsinki and with approval from the Ethics Committee of the China-Japan Friendship Hospital. Written informed consent was obtained from all participants. The diagnosis was made in accordance with guidelines for the prevention and treatment of CHB, published by the Chinese Medical Association in 2010 (18). The criteria for study inclusion were: i) Age, 18–65 years, irrespective of gender; ii) CHB of various degrees in association with liver fibrosis; iii) no intake of medication known to inhibit liver enzymes within two weeks prior to biochemical blood analysis; iv) history of abnormal transaminase; and v) provision of signed informed consent by the patient. The criteria for study exclusion were: i) Unavailability of patient consent; ii) other complicated liver conditions, including other types of viral hepatitis, alcoholic and nonalcoholic fatty liver disease, autoimmune hepatitis and inherited metabolic liver disease; iii) hepatic decompensation, including the presence of ascites; iv) body mass index (BMI) ≥30; v) non-healed upper quadrant abdominal wound; vi) space-occupying tumors or cysts in the right lobe of the liver or various space-occupying tumors and cysts; and vii) acute hepatitis or cholestatic hepatitis.

### LSM

Measurements of TE using FibroScan were performed by a single trained operator. Patients were placed in the supine position with their right arm fully abducted. Measurements were taken from the area over the right lobe of the liver through the intercostal space. At least ten valid TE readings were obtained for each patient and the median value was used for analysis. The results are expressed in kPa. Those cases with a success rate <60% and an interquartile range (IQR)/result ratio >0.3 were regarded as invalid.

### ARFI imaging

Immediately following the FibroScan, the same technician performed a shear wave velocity measurement using ARFI imaging. The right lobe of the liver was localized using a SEQUIOA512 color ultrasound diagnostic system (Siemens Medical Equipment Co., Ltd., Shanghai, China) at a transducer frequency of 5–12 MHz. Ten valid acquisitions were obtained in the region of interest, with the probe positioned 2.5 cm below the skin. All measurements were obtained at the same intercostal space, avoiding large vessels and ribs. The mean, standard deviation (SD) and variation coefficient (SD/mean) of the values from each patient were recorded for statistical analysis.

### FI

Laboratory test results, including tests for hepatitis B, liver function, complete blood count and HBV-DNA were collected. The FI was calculated using the following formula: FI=7.811−3.131×ln[platelet (×10^9^/L)] + 0.781×ln[γ-glutamyl transpeptidase (GGT)] + 3.647 U/L × ln[age (years)]−0.014×cholesterol (mg/dl).

### Liver histology

Liver biopsies were obtained using 16 G or 18 G disposable needles (Bard Peripheral Vascular, Inc., Murray Hill, NJ, USA). Liver biopsy specimens were fixed in formalin, embedded in paraffin and stained with hematoxylin and eosin, silver, Masson Trichrome staining and Sirius Red (all Wuhan Boster Biotechnology, Ltd, Wuhan, China). Necro-inflammatory activity and liver fibrosis was scored according to the biopsy criteria of the Chinese Program of Prevention and Cure for Viral Hepatitis (Tables I and II) (19).

### Statistical analysis

Quantitative variables are expressed as the median (range) and qualitative variables as a percentage. The correlation between the stage of fibrosis and results of the non-invasive tests was assessed using a non-parametric test (Kruskal-Wallis analysis). The diagnostic value of ARFI, FibroScan and FI in predicting significant fibrosis and cirrhosis was assessed by calculating the areas under the respective receiver operator characteristic curves (AUROC). Comparisons of AUROCs were performed according to the Delong method (20). Best cut-off values were determined by optimization of the Younden index, and sensitivity, specificity, as well as positive and negative predictive values (PPV, NPV) were calculated from these same data. For univariate analysis, bivariate Spearman’s rank correlation coefficient was calculated to measure the association between FI, ARFI, FibroScan and other variables, including steatosis. For the subsequent multivariate analysis, a linear regression analysis was performed in order to identify the independent variables influencing the accuracy of the three diagnostic methods. For multiple values, analysis of variance with Chi-squared test was used. Statistical analyses were performed with SPSS, version 17.0 (SPSS, Inc., Chicago, IL, USA). P<0.05 was considered to indicate a statistically significant difference.

## Results

### Patient characteristics

The baseline clinical and biochemical characteristics of the patients are summarized in Table III. The subjects consisted of 71 males (87.7%). The mean age of the patients was 41±11.4 years. The mean BMI was 23±3.0.

### Correlation between stage of fibrosis assessed by biopsy and that measured using non-invasive methods

**Figure 2:**
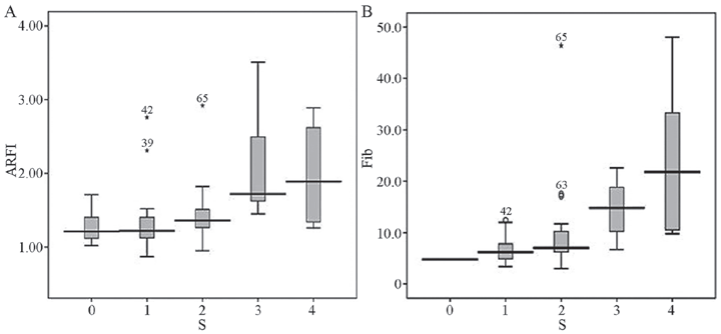
Median ARFI and liver stiffness values in patients with different stages of liver fibrosis. (A) Median ARFI values in patients with S0, S1, S2, S3 and S4 were 1.28, 1.33, 1.43, 2.07 and 1.98 m/s, respectively. (B) Median liver stiffness values in patients with S0, S1, S2, S3 and S4 were 4.8, 6.69, 9.87, 14.8 and 24.2 kPa, respectively. Upper horizontal line, maximum value beside outlier; lower horizontal line, the minimum value; middle horizontal line, median; box, ¼–3/4 of values; star/circle/dot, outliers. ARFI, acoustic radiation force impulse elastography; Fib, FibroScan.

A significant association was identified between the stage of fibrosis and the values obtained by ARFI, FibroScan and FI. The median values of ARFI according to fibrosis stage were 1.28±0.21, 1.33±0.32, 1.43±045, 2.07±0.61 and 1.98±0.52 m/s for S0–S4, respectively, while the median FibroScan measurements were 4.8±0.01, 6.69±0.23, 9.87±2.11, 14.8±3.24 and 24.2±5.11 kPa for S0–S4, respectively, and the mean FI scores in patients with S0, S1, S2, S3 and S4 stages of liver fibrosis were 6.75±1.17, 6.21±1.38, 7.23±2.25, 8.68±1.71 and 10.31±1.58, respectively (Fig. 2).

The normal distribution using Pearson’s correlation and the partial distribution using the Spearman’s correlation demonstrated that the three diagnostic methods significantly correlated with the stage of fibrosis [ARFI (r=0.577, P<0.001); FibroScan (r=0.629, P<0.001); and FI (r=0.539, P<0.001)].

### Diagnosis of S≥2 and cirrhosis using individual and combinations of methods

**Figure 3:**
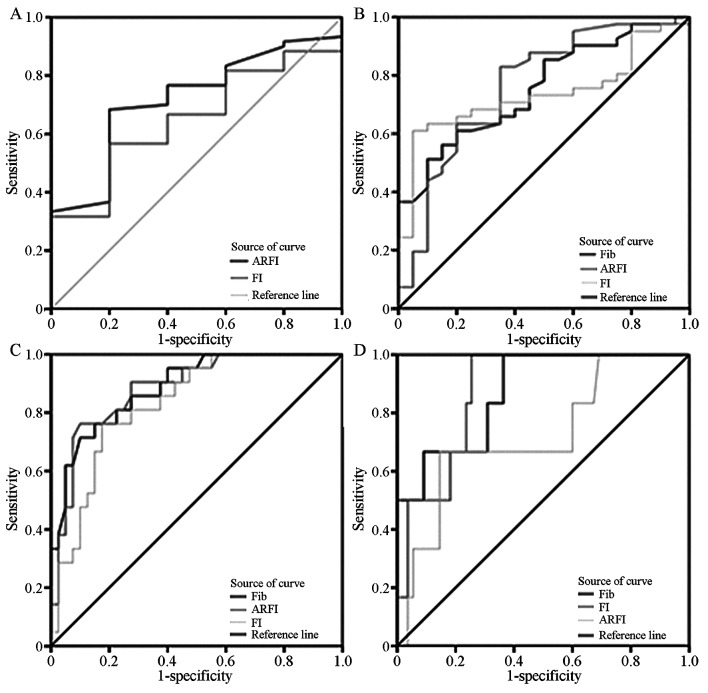
Diagnostic performance of ARFI, FibroScan and FI for the different histological stages of fibrosis. (A) ROC of ARFI, FibroScan and FI for the diagnosis of histological stages ≥S1; (B) ROC of ARFI, FibroScan and FI for the diagnosis of histological stages ≥S2; (C) ROC of ARFI, FibroScan and FI for the diagnosis of histological stages ≥S3; (D) ROC of ARFI, FibroScan and FI for the diagnosis of histological stage S4. ARFI, acoustic radiation force impulse elastography; Fib, FibroScan; FI, Forns’ index; ROC, receiver operator curve.

In order to evaluate the power of ARFI, FibroScan and FI to accurately predict the stage of fibrosis in this population of patients, an ROC analysis was performed. This analysis revealed that the AUROCs of ARFI, FibroScan and FI compared with the stage of fibrosis, as determined by liver biopsy, were 0.790±0.084, 0.838±0.074 and 0.814±0.721, respectively, for S≥2. Table IV and Fig. 3 depict the AUROCs and show the diagnostic performance of the three methods.

**Figure 4:**
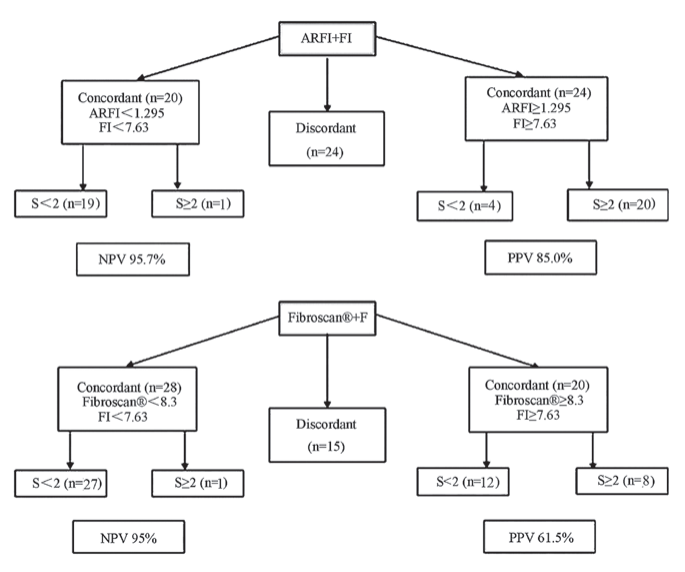
Flowchart of the synchronous application of FI with either ARFI or FibroScan^®^ for the diagnosis of S≥2. ARFI, acoustic radiation force impulse elastography; FI, Forns’ index; NPV, negative predictive value; PPV, positive predictive value.

When the methods were combined using the cut-offs shown in Table IV, the PPV for the joint use of FI and ARFI for the diagnosis of S≥2 was 85.0%, while the NPV was 95.7%. Similarly, the PPV and NPV for the combination of FibroScan and FI were 61.5 and 95.0%, respectively, for S≥2 (Table V, Fig. 4).

### Inflammation is correlated with fibrosis

ARFI measurements were 1.21, 1.25, 1.41, 1.71 and 2.4 m/s for inflammation grades G0, G1, G2, G3 and G4, respectively (P=0.005). FibroScan measurements were 4.8, 6.8, 7.8, 13.4 and 22.6 kPa for inflammation grades G0, G1, G2, G3 and G4, respectively (P=0.002). Finally, for FI they were 6.76±1.17, 6.48±1.58, 7.61±2.22, 8.82±2.29 and 9.51±2.18 for inflammation grades G0, G1, G2, G3 and G4, respectively (P=0.034). There was no significant different between G0 and G1 as well as between G2, G2 and G3, although there were statistically significant differences between G0 and G3 as well as G1 and G3. Spearman’s correlation test was performed in order to identify whether inflammation altered the prediction of fibrosis by the various diagnostic methods. There was no significant correlation between the degree of inflammation and the stage of fibrosis.

In order to assess the influence of hepatic inflammation on the ARFI, FibroScan and FI scores, patients with liver fibrosis and significant inflammation (G2 or higher) were compared with patients with fibrosis but without inflammation (G0). In this analysis, no significant correlation between ARFI, FibroScan and FI results, with inflammation grades was detected (Table VI).

### Influence of steatosis on prediction of fibrosis

The effects of concomitant steatosis on the results obtained by the different diagnostic methods were investigated. Therefore, all patients were divided into one group with hepatitis B and fatty liver and another group with uncomplicated hepatitis B. No statistically significant differences were detected between the two groups (P-values were 0.403, 0.162 and 0.200, respectively).

Univariate correlation analysis was performed between ARFI, FibroScan and FI, and other variables, including steatosis. The stage of fibrosis was the variable most significantly correlated with ARFI, FibroScan and FI scores. Platelet count, prothrombin time, activity grade and γ-glutamyl transpeptidase levels were also significantly correlated with ARFI, FibroScan and FI, while the presence of steatosis was not (Table VII).

Table VIII shows the results of the linear regression analysis used to identify the independent variables influencing ARFI, FibroScan and FI. Steatosis failed to show a statistically significant effect.

## Discussion

Liver biopsy is currently the gold standard used to determine the stage of liver fibrosis, with the results being used to assess the disease stage as well to decide on the appropriate therapy (9). However, a biopsy is an invasive test, which requires the patient to be hospitalized and is associated with certain risks, including pain and bleeding. In addition, liver biopsies are more expensive than noninvasive tests and the results are subject to sampling errors. A further limitation of liver biopsy is that different pathologists may interpret the same sample differently, which may result in discrepancies in disease staging. Therefore, noninvasive tests have recently been developed. ARFI and TE (FibroScan) are rapid techniques with highly reproducible results that may be used for measuring liver tissue stiffness. A number of studies have demonstrated the accuracy of these methods in assessing the degree of hepatic fibrosis (17,21).

The FI is based on platelet count, GGT, age and cholesterol. The presence of significant fibrosis has been shown to be predicted with a 96% negative predictive value (NPV) and a 66% positive predictive value (PPV) (22). Novel scores or biomarkers have been used to improve the prediction of fibrosis and may help to detect severe fibrosis, although they lack sensitivity and specificity (20,23). Therefore, it is probable that a combination of different non-invasive markers may be required to ensure accurate diagnoses. Boursier *et al* (21) have suggested that a combination of LSE and blood tests for fibrosis may improve diagnostic accuracy in patients with chronic hepatitis C. However, their study did not evaluate the statistical differences between the area under the receiver operating characteristic curves, which is the only diagnostic index used for fibrosis tests and their combination. Furthermore, in clinical practice these methods assessing liver elasticity may be effected by a number of factors; predominantly by hepatic fibrosis, but also by necrosis-inflammatory activity (16), body mass index (24), steatosis (25) and extrahepatic cholestasis (26,27).

In the present study, measurements obtained by ARFI and FibroScan as well as FI scores were significantly correlated with the stage of fibrosis in patients with hepatitis B, as assessed by liver biopsy, which suggested that these noninvasive diagnostic methods were adequate for evaluation of the stage of liver fibrosis.

Recent studies have suggested that the combination of serum markers with FibroScan is highly accurate in the identification of liver fibrosis (28). In the present study, the combination of FI with either FibroScan or ARFI increased the PPV and NPV of any of the tests individually and provided reliable identification of significant fibrosis and cirrhosis in a large proportion of patients. These results suggested that a large number of patients with liver fibrosis may be diagnosed and staged without any biopsy required.

A number of factors affect liver stiffness. Studies have shown that inflammation activity can alter the LSM value (29). Coco *et al* (30) reported that liver stiffness increased 1.3- to 3-fold following temporary increases in the levels of alanine transaminase, but that it decreased to baseline values thereafter. The same study demonstrated that liver stiffness was significantly different in patients with hepatitis inflammation in comparison with patients with stable biochemical markers. It was postulated that the inflammatory infiltrate and edema may have had an impact on the TE value (31).

In the present study, the results of assessment using ARFI, FibroScan and FI were significantly different depending on the grade of inflammation. Further comparison of the interclass groups G0 and G1 as well as G2, G2 and G3 demonstrated no significant differences, although there were statistically significant differences between G0 and G3, and between G1 and G3. It may be that inflammatory activity stimulates the activation and proliferation of hepatic stellate cells, thus increasing the levels of collagen I and III.

Hepatic steatosis may be another factor that influences liver stiffness values. Fatty tissues are softer than healthy liver parenchyma, which reduces liver stiffness (30,32). A number of studies have investigated the impact of steatosis on liver stiffness. Sandrin *et al* (33) reported that elasticity measurements were correlated with the stage of fibrosis only and not with necro-inflammatory activity or steatosis grades in patients with chronic hepatitis C. A recent study conducted on healthy subjects suggested that liver stiffness values are not influenced by steatosis (34). In the present study, no significant difference in elasticity was detected between patients with hepatitis B who had fatty liver and those who did not. Furthermore, in the univariate analysis, steatosis had no influence on the results of the three methods of diagnosis in patients with CHB. There are naturally limitations to the present study: The ALT levels were not considered, and as ALT was recently shown to be a significant factor influencing LSM (33), the results of the present study may be misleading. Furthermore, the results are based on a small sample size; the number of patients with fatty liver was only 30. Thus, larger, multicenter studies are required to confirm or refute these findings.

In conclusion, ARFI, FibroScan and FI were proven to be reliable methods with which to assess fibrosis in patients with hepatitis B. Indeed, the combined use of FI with either ARFI or FibroScan appears to be a promising approach, which may increase the diagnostic accuracy of these tests individually. In addition, combining these approaches resolves the majority of discordant results between non-invasive tests and improves the reliable individual diagnosis for significant fibrosis and cirrhosis, thus reducing the requirement for liver biopsies. Inflammatory activity may influence the diagnostic value of these methods to a certain extent. However, steatosis did not produce a significant impact on the diagnostic values in patients with CHB. The methods evaluated in the present study are an ideal tool for diagnosis and detecting changes in the stage of fibrosis and may therefore be useful for monitoring disease progression and regression, as well as in predicting clinical outcomes in the future.

## Figures and Tables

**Table I:** Criteria for the grading of chronic hepatitis.

Grading	Portal tract and periportal inflammation	Lobular inflammation
0	Absent	Absent
1	Portal inflammation	Degeneration and few potty, focal necrosis
2	Mild piecemeal necrosis	Degeneration, spotty, focal necrosis or acidophilic body
3	Moderate piecemeal necrosis	Degeneration, confluent necrosis or bridging necrosis
4	Severe piecemeal necrosis	Widely bridging necrosis, involved multiple lobule (multiple lobule necrosis)

**Table II:** Criteria for the staging of chronic hepatitis.

Staging	Degree of fibrosis
0	Absent
1	Portal fibrosis to be enlarged, localized perisinusoidal and intralobular fibrosis
2	Periportal fibrosis, several fibrous septa with lobule structure remaining
3	Numerously fibrous septa companied, Lobule structure distortion, without cirrhosis
4	Early cirrhosis

**Table III:** Baseline characteristics (n=81).

Characteristic	Fibrosis stage, n (%)					
	Total	S0	S1	S2	S3	S4
Male gender	71 (87.7)	2 (22.2)	21 (84.0)	24 (96.0)	13 (81.2)	8 (100)
Age (years)	41±11.4	38±12.3	38±10.2	40±11.9	46±12.0	48±6.2
Steatosis	30/74	0/1	11/25	7/24	9/16	3/8
(n/total)						
Inflammation	–	9 (G0:9 G1:0 G2:0 G3:0 G4:0)	25 (G0:0 G1:11 G2:14 G3:0 G4:0)	25 (G0:0 G1:5 G2:14 G3:6 G4:0)	16 (G0:0 G1: 3 G2:7 G3:5 G4:1)	8 (G0:0 G1: 1 G2:5 G3:2 G4:0)
BMI	23±3.0	22±1.9	23±2.0	24±3.5	23±3.3	25±3.6
GGT	58±66.5	98±42.9	36±7.4	41±7.3	68±14.8	129±56.3
PLT	176±63.4	199±35.3	205±46.9	182±71.3	140±58.7	112±56.6
TBIL	17±21.9	41±24.0	15±1.4	13±0.8	17±1.7	16±4.4
CHO	6±12.6	5±0.4	10±5.0	5±0.2	4±0.2	5±0.5
ALP	78±35.1	105±58.3	64±11.6	66±17.0	85±27.4	85±14.6
PT (INR)	1±0.1	0.9±0.1	1.0±0.1	1.0±0.1	1.0±0.1	1.1±0.1
ALB	44±9.4	46±2.3	43±14.1	46±3.5	43±9.4	43±2.9

BMI, body mass index; GGT, γ-glutamyl transpeptidase; PLT, platelet count; TBIL, bilirubin; CHO, cholesterol; ALP, alkaline phosphatase; PT(INR), prothrombin time (international normalized ratio; ALB, albumin.

**Table IV:** Diagnostic performance of ARFI, FibroScan and FI for the diagnosis of different histological stages.

Variable	Mode	≥S1	≥S2	≥S3	S4
Cut-off	ARFI	1.295	1.295	1.54	1.835
	Fib	–	10.3	11.85	9.4
	FI	6.82	7.55	7.902	8.45
AUROCs	ARFI	0.720	0.762	0.884	0.723
	Fib	0	0.753	0.888	0.873
	FI	0.650	0.735	0.832	0.876
95%CI	ARFI	0.524–0.916	0.627–0.896	0.798–0.970	0.501–0.944
	Fib	–	0.631–0.875	0.805–0.970	0.740–1.006
	FI	0.452–0.828	0.610–0.861	0.731–0.933	0.771–0.981
Se (%)	ARFI	68.3	82.9	76.2	66.7
	Fib	–	51.2	71.4	100
	FI	56.7	61.0	76.2	100
Sp (%)	ARFI	80.0	65.0	90.0	85.5
	Fib	–	90.0	90.0	63.6
	FI	80.0	95.0	82.5	74.5

ARFI, acoustic radiation force impulse elastography; FI, Forns’ index; Se, sensitivity; Sp, specificity; CI, confidence interval.

**Table V:** Diagnostic performance of FI with either ARFI or FibroScan for the diagnosis of S≥2.

Mode	PPV	NPV	FPR (%)	FNR (%)	Se (%)	Sp (%)	Accuracy
ARFI + FI	85.0	95.7	12.0	5.6	94.4	88.0	90.7
Fib + FI	61.5	95	34.5	5.9	94.1	65.5	76.1

FI, Forns’ index; ARFI, acoustic radiation force impulse elastography; PPV, positive predictive value; NPV, negative predictive value; FPR, false positive rate; FNR, false negative rate; Fib, FibroScan.

**Table VI:** Diagnostic performance of ARFI, FibroScan and FI for G<2 and G≥2.

Stage	Mode	G<2	G≥2	P-value
S1	ARFI	1.13	1.285	0.201
	Fib	6.7	6.15	0.688
	FI	6.14±1.88	6.27±1.57	0.841
S2	ARFI	1.36	1.41	0.406
	Fib	6.5	8.15	0.227
	FI	6.09±1.81	7.59±2.30	0.199
S3	ARFI	1.5	2.085	0.136
	Fib	7.7	16.85	0.101
	FI	8.16±1.65	8.80±1.78	0.579

ARFI, acoustic radiation force impulse elastography; FI, Forns’ index; Fib, FibroScan.

**Table VII:** Univariate correlation analysis between ARFI, FibroScan, Forns’ index and other variables.

Variable	FibroScan		ARFI		Forns’ index	
	Correlation coefficient	P-value	Correlation coefficient	P-value	Correlation coefficient	P-value
Fibrosis stage	0.629	<0.001	0.577	<0.001	0.528	<0.001
Platelet count	−0.380	0.001	−0.359	0.001	−0.803	<0.001
Prothrombin time	0.347	0.004	0.259	0.025	0.359	0.002
Albumin	−0.239	0.074	−0.377	0.003	−0.219	0.092
Age	0.202	0.094	0.202	0.094	0.202	0.094
Bilirubin	0.250	0.039	0.181	0.118	0.325	0.004
Activity grade	0.451	<0.001	0.441	<0.001	0.337	0.004
Body mass index	0.300	0.014	0.005	0.965	0.100	0.407
Gender	−0.012	0.920	−0.012	0.920	−0.012	0.920
HbeAg positivity	0.040	0.743	0.040	0.743	0.040	0.743
Alkaline phosphatase	0.294	0.015	0.414	<0.001	0.106	0.365
γ-glutamyl transpeptidase	0.680	<0.001	0.338	0.004	0.460	<0.001
Steatosis score	0.170	0.163	−0.100	0.407	0.154	0.216

ARFI, acoustic radiation force impulse elastography; HbeAg, hepatitis B e antigen.

**Table VIII:** Multivariate analysis toward predicting ARFI, FibroScan and Forns’ index.

Variable	FibroScan		ARFI		Forns’ index	
	Estimate	P-value	Estimate	P-value	Estimate	P-value
Fibrosis stage	0.384	0.001	0.042	0.768	0.179	0.047
Steatosis	0.139	0.108	−0.062	0.559	0.111	0.128
Platelet	0.006	0.951	−0.084	0.131	−0.675	<0.001
Prothrombin time	0.088	0.319	0.282	0.017	0.069	0.384
Bilirubin	0.120	0.146	–	–	0.106	0.156
Activity grade	−0.043	0.629	0.112	0.332	−0.046	0.575
Body mass index	0.116	0.180	–	–	–	–
Alkaline phosphatase	0.017	0.844	0.339	0.005	–	–
γ-glutamyl transpeptidase	0.497	<0.001	0.399	0.002	0.108	0.182

ARFI, acoustic radiation force impulse elastography.
